# Phosphate handling as a determinant of osteoporosis in primary hyperparathyroidism

**DOI:** 10.1530/EC-26-0118

**Published:** 2026-04-21

**Authors:** Sebastian Szewczyk, Michał Popow, Urszula Ambroziak

**Affiliations:** ^1^ Department of Internal Medicine and Endocrinology, Medical University of Warsaw, Warsaw, Poland; ^2^ Doctoral School of Medical University of Warsaw, Warszawa, Poland

**Keywords:** primary hyperparathyroidism, parathyroid hormone, osteoporosis, calcitriol, phosphate metabolism, vitamin D, biomarkers

## Abstract

**Introduction:**

Primary hyperparathyroidism is characterized by chronic parathyroid hormone excess, leading to hypercalcemia, increased bone turnover, and skeletal complications. Although osteoporosis is a common manifestation of these, the biochemical determinants of bone loss remain insufficiently defined. The roles of active vitamin D and renal phosphate handling require further clarification. This study aimed to identify biochemical determinants of osteoporosis in patients with primary hyperparathyroidism, with a particular focus on the contribution of calcitriol levels and renal phosphate handling. We further sought to evaluate their predictive performance in discriminating osteoporotic from non-osteoporotic individuals.

**Materials and methods:**

We retrospectively analyzed 74 adults with primary hyperparathyroidism ineligible for surgery, assessing serum calcium, phosphate, vitamin D metabolites, parathormone, and 24 h urinary calcium. Renal phosphate handling was estimated by TMP/GFR. Logistic regression and ROC analyses identified independent predictors and optimal cutoff values for osteoporosis.

**Results:**

Osteoporosis was present in 33.8% of patients. Individuals with osteoporosis demonstrated significantly higher calcitriol levels and lower renal phosphate reabsorption, also in multivariate analysis, while serum calcium, phosphate, and 25-hydroxyvitamin D did not differ between groups. Receiver operating characteristic curve analysis identified clinically meaningful cutoff values for both parameters.

**Conclusion:**

Increased levels of the active form of vitamin D and impaired renal conservation of phosphate are independently associated with osteoporosis in primary hyperparathyroidism, outperforming traditional biochemical markers. Incorporating these measures into routine clinical assessment may improve identification of patients at high skeletal risk and enhance decision-making in the management of bone disease in primary hyperparathyroidism.

## Introduction

Primary hyperparathyroidism (PHPT) is one of the most common endocrine disorders and is characterized by inappropriate, autonomous secretion of parathyroid hormone (PTH) by one or more parathyroid glands. It is the most frequent cause of hypercalcemia in the general population (1). Recent data indicate that the prevalence of PHPT increased from 0.84 to 1.02%, with higher rates observed in women, although the trend suggests a tendency toward stabilization (2). The symptoms and complications of hyperparathyroidism are related to the actions of parathyroid hormone on target tissues, leading to elevated serum calcium levels. PTH stimulates renal reabsorption of calcium, suppressing renal reabsorption of phosphate, increasing bone resorption, and stimulating intestinal calcium absorption by increasing the production of active form of vitamin D – calcitriol – in the proximal renal tubule (1).

Osteoporosis, alongside nephrolithiasis, is one of the most common skeletal complications of PHPT, although reported prevalence varies widely depending on population characteristics and diagnostic criteria (3). In the literature, older age, hip osteoporosis, a history of previous fractures, and reduced renal function were identified as potential risk factors for osteoporosis (4). The mechanisms underlying bone loss in PHPT are multifactorial, but still underestimated. Sustained PTH excess leads to continuous stimulation of osteoblasts and osteocytes to express receptor activator of nuclear factor-κB ligand (RANKL), thereby promoting osteoclast differentiation and increased bone resorption. Simultaneously, PTH enhances renal tubular calcium reabsorption and phosphate excretion while activating 1α-hydroxylase, which increases calcitriol synthesis. Elevated calcitriol further increases intestinal calcium absorption but may also contribute to bone resorption by amplifying calcium mobilization and suppressing PTH feedback inhibition (5). The combined effects of enhanced bone turnover, negative phosphate balance, and increased calcium release from the skeleton lead to progressive cortical bone loss characteristic of PHPT-related osteoporosis. Although PTH-mediated bone loss is well established, the specific role of renal phosphate handling, vitamin D metabolism, and other biochemical determinants in modulating bone density in PHPT remains poorly characterized.

Osteoporosis represents a significant clinical concern in Poland, affecting an estimated 2.1 million individuals – 1.7 million of whom are women – according to 2023 data from the National Health Fund (NFZ) (6), making it one of the most prevalent diseases in the country. Notably, up to 75% of patients remain undiagnosed and untreated. Identifying risk factors that may predispose individuals to osteoporosis and facilitate early detection is very important, as their recognition could accelerate diagnostic evaluation and therapeutic intervention.

The present study aimed to assess the relationship between calcium–phosphate metabolism parameters and the occurrence of osteoporosis in patients with PHPT.

## Materials and methods

This was a retrospective, single-center observational study. We included 74 adult patients (68 women and 6 men) with a confirmed diagnosis of PHPT who were managed at our department between 2022 and 2025. The diagnosis of PHPT was established based on the presence of persistent hypercalcemia (total and/or ionized calcium) with inappropriately elevated or non-suppressed parathyroid hormone (PTH) levels, after exclusion of secondary causes (1). No additional sub-selection beyond the predefined inclusion criteria was applied. Clinical, biochemical, and densitometric data were retrieved from the CGM-CLININET electronic clinical information system, which is routinely used and maintained by our department for inpatient and outpatient care. Laboratory results and bone mineral density (BMD) measurements were extracted directly from the system and verified against original reports available in the hospital medical records. All data were anonymized at the time of extraction and stored in a dedicated database for subsequent analysis; the extracted variables were manually cross-checked against original laboratory and densitometric reports to minimize data entry and transcription errors.

Inclusion criteria for the study included the following:

— age over 18 years,
— diagnosis of PHPT, and
— non-surgical management of PHPT at study entry.

The main exclusion criteria were as follows:

— secondary or tertiary hyperparathyroidism,
— 25-hydroxyvitamin D [25(OH)D] level below 20 ng/mL,
— chronic kidney disease stage 3 or higher according to Kidney Disease: Improving Global Outcomes (KDIGO) classification (7),
— pregnancy or breastfeeding, and
— subjects with known genetic disorders affecting bone metabolism (e.g., familial hypocalciuric hypercalcemia and multiple endocrine neoplasia).

Serum levels of calcium, magnesium, alkaline phosphatase (ALP) and phosphorus, and 25(OH)D were measured using colorimetric assays on Roche Cobas and Siemens Dimension EXL analyzers (Roche Diagnostics, Germany; Siemens Healthineers, Germany). Serum calcium values were corrected for albumin concentration using the Payne formula. Serum magnesium and calcitriol levels were assessed selectively in patients with hypercalcemia of initially unclear etiology, reflecting the tertiary referral setting of our center. Magnesium measurement aided differentiation between PHPT and familial hypocalciuric hypercalcemia by supporting interpretation of calcium–PTH set-point abnormalities (8). PTH concentrations were determined using a second-generation electrochemiluminescence immunoassay (ECLIA) on a Roche Cobas e601 analyzer (Roche Diagnostics, Germany). Calcitriol (1,25-dihydroxyvitamin D) was measured to identify or exclude calcitriol-mediated hypercalcemia and other disturbances of vitamin D metabolism when PTH levels were not clearly diagnostic (9). It was measured by radioimmunoassay (RIA) on a Diasorin LIAISON® XL platform (Diasorin S.p.A., Italy), with a functional sensitivity of 10 ng/mL and an inter-assay coefficient of variation (CV) of less than 8%. 24 h urinary calcium concentration and single (spot) urine sample were assessed using colorimetric methods on Roche Cobas and Siemens Dimension EXL analyzers. Urinary creatinine levels were measured using the compensated kinetic Jaffe method on the Roche Cobas system (Roche Diagnostics, Germany). All urine samples were stored at 4°C until analysis. All assays were performed according to the manufacturers’ instructions. To avoid interpretive errors due to differences in urine volume or individual factors, such as weight, muscle mass, or age, the results were normalized per gram of creatinine. Hypercalciuria was defined as urinary calcium excretion >6.25 mmol/24 h in women and >7.5 mmol/24 h in men. Tubular maximum reabsorption of phosphate per glomerular filtration rate (TMP/GFR) was used to assess renal phosphate handling, as it provides a more accurate and physiologically relevant measure of renal phosphate reabsorption than isolated urinary phosphate excretion. Unlike simple phosphate excretion, TMP/GFR accounts for variations in serum phosphate concentration and glomerular filtration rate, allowing for better characterization of phosphate metabolism in PHPT (10). TMP/GFR was collected from a single urine sample and calculated using the Walton–Bijvoet formula:

TMP/GFR = Ppi − (SCr × UPi)/UCr),

where

TMP/GFR – tubular maximum for phosphate reabsorption per glomerular filtration rate,

Ppi – plasma inorganic phosphate concentration (mg/dL),

SCr– serum creatinine concentration (mg/dL),

UPi – urine inorganic phosphate concentration (mg/dL), and

UCr– urine creatinine concentration (mg/dL).

Osteoporosis was defined according to WHO criteria as a T-score ≤ −2.5 at the lumbar spine, femoral neck, or total hip on DXA in postmenopausal women and men aged ≥ 50 years. In younger individuals (<40 years), BMD was compared to the reference range for age and gender. Statistical analyses were performed using STATISTICA software (version 13.3.721.0, 64-bit; StatSoft, Poland). Continuous variables are presented as mean ± standard deviation (SD). The distribution of each variable was assessed using the Shapiro–Wilk test. For normally distributed data, comparisons between dependent variables were performed using the paired Student’s *t*-test, whereas the Wilcoxon signed-rank test was applied for non-normally distributed variables (*P* ≤ 0.05 in the Shapiro–Wilk test). Changes in biochemical parameters over time were compared using the Student’s *t*-test or Wilcoxon test, as appropriate. Univariate logistic regression analyses were conducted to evaluate the relationship between individual study variables and the presence of osteoporosis (dependent variable coded as 1 = present; 0 = absent). Variables with a significance level of *P* < 0.20 in the univariate analysis were entered into a multivariate logistic regression model to identify independent predictors of osteoporosis (Table 1). For each variable, odds ratios (ORs) with 95% confidence intervals (CIs) were calculated to estimate the relative odds of osteoporosis per unit increase in the predictor. Receiver operating characteristic (ROC) curve analysis was used to assess the discriminatory power of selected biochemical markers. The Youden index was applied to determine optimal cutoff points associated with an increased risk of osteoporosis. Sensitivity and specificity at the optimal cutoff points were reported with corresponding 95% CIs. Outliers were identified graphically and excluded if they fell outside three interquartile ranges (IQRs). Missing data were handled using listwise deletion. Multivariate logistic regression models were limited to variables with adequate event counts to avoid model overfitting (Table 2).

**Table 1:** Correlations between biochemical parameters and clinical outcomes in patients with primary hyperparathyroidism.

Variables compared	*r*	*P*	Direction
PTH – calcitriol	0.37	<0.05	Positive
PTH – TMP/GFR	−0.46	<0.05	Negative
PTH – 25(OH)D	−0.08	>0.05	None
PTH – osteoporosis	−0.03	>0.05	None
PTH – calcium	0.41	<0.05	Positive
PTH – phosphorus	−0.48	<0.05	Negative
Calcitriol – 25(OH)D	0.03	>0.05	None
Calcitriol– TMP/GFR	−0.30	<0.05	Negative
Calcitriol – 24 h urinary calcium excretion	0.37	<0.05	Positive
Calcitriol – osteoporosis	0.02	>0.05	None
Osteoporosis – 24 h urinary calcium excretion	−0.25	<0.05	Negative
Osteoporosis – GFR	0.50	<0.05	Positive
Osteoporosis – serum calcium	0.24	<0.05	Positive
Cl/Pi ratio – PTH	0.48	<0.05	Positive
Cl/Pi ratio – calcitriol	0.35	<0.05	Positive
Cl/Pi ratio – TMP/GFR	−0.91	<0.05	Negative
Age – osteoporosis	0.30	<0.05	Positive
Age – GFR	−0.66	<0.05	Negative

**Table 2:** Multivariate logistic regression analysis for predictors of osteoporosis.

Variable	*β* coefficient	SE (standard error)	Wald *χ*^2^	OR (95%CI)	*P*-value
Calcitriol (pg/mL)	0.076	0.030	6.60	1.08 (1.02–1.14)	0.010
TMP/GFR	−8.842	4.227	4.38	0.41 (0.18–0.95)	0.036
Parathormone (pg/mL)	−0.002	0.014	0.02	0.99 (0.97–1.02)	0.88
Age (years)	0.032	0.027	1.92	1.03 (0.97–1.09)	0.41

## Results

**Figure 1:**
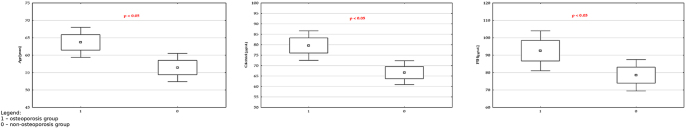
Comparison of age, calcitriol and PTH concentration between the two groups (Group 1 – osteoporosis group; Group 0 – non-osteoporosis group).

**Figure 2:**
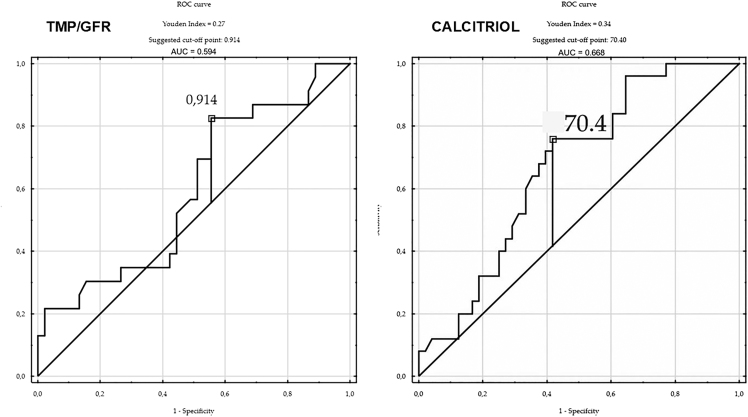
Receiver operating characteristic (ROC) curves for serum calcitriol and TMP/GFR in predicting osteoporosis in patients with PHPT.

The baseline clinical and biochemical characteristics of the study participants are summarized in Table 3. The study cohort consisted of 74 adults (91.9% women; 8.1% men) with a median age of 59 years (range: 19–80 years). Patients with osteoporosis were significantly older than those without osteoporosis (median: 64 vs 56.5 years, *P* = 0.03). Nephrolithiasis was observed exclusively in the osteoporosis group (28.8% of the total cohort). Regarding biochemical parameters, age, serum PTH and calcitriol concentrations were significantly higher in patients with osteoporosis compared to those without (as is shown in Fig. 1). In contrast, serum 25(OH)D, calcium, magnesium, and phosphate concentrations did not differ significantly between the groups. PTH, calcitriol, and age were positively correlated with the osteoporosis. The mean serum calcium concentration, corrected for albumin according to the Payne formula, was within the hypercalcemic range for both groups. Urinary parameters, including 24 h calcium excretion and TMP/GFR, also showed no statistically significant intergroup differences. However, there was a trend toward lower TMP/GFR in patients with osteoporosis (0.83 ± 0.15 vs 0.89 ± 0.16, *P* = 0.19), suggesting a possible reduction in tubular phosphate reabsorption. Serum PTH levels correlated positively with both calcitriol (*r* = 0.37, *P* < 0.05) and total serum calcium (*r* = 0.41, *P* < 0.05), while showing inverse correlations with TMP/GFR (*r* = −0.46, *P* < 0.05) and serum phosphate (*r* = −0.48, *P* < 0.05). There were no significant associations between PTH and 25(OH)D (*r* = −0.08, *P* > 0.05) or between PTH and the presence of osteoporosis (*r* = −0.03, *P* > 0.05). Calcitriol levels were unrelated to 25(OH)D (*r* = 0.03, *P* > 0.05) but correlated inversely with TMP/GFR (*r* = −0.30, *P* < 0.05) and positively with 24 h urinary calcium excretion (*r* = 0.37, *P* < 0.05). No correlation was observed between calcitriol and osteoporosis status (*r* = 0.02, *P* > 0.05). Osteoporosis was weakly associated with 24 h urinary calcium excretion and calcium and GFR. The Cl/Pi ratio did not differ between patients with and without osteoporosis but correlated positively with PTH and calcitriol and strongly inversely with TMP/GFR (*r* = −0.91). In multivariate logistic regression analysis, higher serum calcitriol levels were independently associated with an increased risk of osteoporosis (*β* = 0.076, OR: 1.08, 95% CI: 1.02–1.14, *P* = 0.010), whereas higher fasting TMP/GFR values were inversely related to osteoporosis risk (*β* = −8.842, OR: 0.41, 95% CI: 0.18–0.95, *P* = 0.036). Neither PTH nor age retained statistical significance in the multivariate model. ROC curve analysis identified a calcitriol cutoff value of 70.4 pg/mL, above which the likelihood of osteoporosis increased markedly. Conversely, a TMP/GFR threshold of 0.914 was determined as the lower limit associated with higher osteoporosis risk (as shown in Fig. 2).

**Table 3:** Characteristics of the patients and correlation with osteoporosis incidence. (Bold values indicate statistically significant differences (*P* < 0.05).

Characteristics	Normal values	Whole group	Osteoporosis		*P*	*r* ^†^
			(+): *n* = 25	(−): *n* = 48		
Age		59 (19–80)	64	56.5	**0.03**	**0.25**
Sex						
Males		6 (8.1%)				
Females		68 (91.9%)				
Nephrolithiasis		21 (28.8%)	21	0	**0.00**	0.11
Calcium, mmol/L*	2.2–2.6	2.64 ± 0.16	2.68 ± 0.21	2.61 ± 0.19	0.63	−0.02
Magnesium, mmol/L	0.75–1	0.88 ± 0.07	0.85 ± 0.10	0.91 ± 0.04	0.81	0.08
Phosphorus, mmol/L	0.8–1.45	0.72 ± 0.14	0.73 ± 0.17	0.68 ± 0.13	0.73	0.01
Chloride, mmol/L	97–110	105.58 ± 2.48	105.82 ± 2.64	105.12 ± 2.12	0.25	−0.14
25(OH)D, ng/mL	30–50	31.7 ± 7.41	28.47 ± 11.65	30.32 ± 10.59	0.39	0.11
Parathyroid hormone, pg/mL	15–65	83.4 ± 29.8	92.5 ± 28.22	78.4 ± 29.79	<0.05	0.23
Calcitriol, pg/mL		71.07 ± 20.3	85.23 ± 30.47	72.79 ± 28.94	<0.05	0.30
Creatinine, mg/dL	0.5–1	0.75 ± 0.12	0.76 ± 0.13	0.79 ± 0.11	0.40	0.10
Alkaline phosphatase, U/L	30–120	89.06 ± 26.55	80.37 ± 22.89	107.11 ± 24.77	**<0.05**	0.20
24 h urinary calcium excretion, mmol/24 h		8.01 ± 3.24	8.96 ± 3.80	7.98 ± 3.97	0.20	−0.10
TMP/GFR single urine collection		0.87 ± 0.15	0.83 ± 0.15	0.89 ± 0.16	0.19	−0.15
Cl/Pi^‡^ ratio		115.92 ± 24.30	116.18 ± 20.32	115.79 ± 26.34	0.94	0.25

* Serum calcium concentrations were adjusted for albumin levels using the Payne formula.

^†^ Correlation with osteoporosis.

^‡^ Chloride/phosphorus serum ratio.

## Discussion

In this study, we investigated the relationship between parameters of calcium–phosphate metabolism and the occurrence of osteoporosis in patients with PHPT. Higher serum calcitriol levels and lower TMP/GFR were independently associated with osteoporosis; however, these parameters represent integrated downstream readouts of sustained PTH activity rather than separate pathogenic mechanisms. The lack of a direct association between circulating PTH levels and osteoporosis likely reflects the pulsatile secretion and short half-life of PTH, which limit the ability of single-time-point measurements to capture chronic biological exposure, whereas calcitriol levels and renal phosphate handling more accurately reflect long-term PTH effects relevant to skeletal involvement.

Although links between vitamin D metabolism and bone health have long been proposed, direct evidence that elevated calcitriol *per se* causes bone loss in humans is lacking. In PHPT, circulating calcitriol primarily reflects PTH-driven renal 1α-hydroxylase activation and appears to be associated with skeletal involvement as an integrated marker of sustained PTH activity rather than an independent pathogenic factor (11, 12). In contrast, the inactive precursor 25(OH)D shows limited and clinically modest associations with skeletal outcomes in PHPT, with no significant effects on volumetric bone density, microarchitecture, or bone strength after adjustment for demographic factors (13, 14). Previous research has primarily focused on the therapeutic role of calcitriol in the management of postmenopausal osteoporosis. Early clinical trials (15) suggested that calcitriol supplementation might improve BMD and reduce fracture risk compared with calcium alone. However, subsequent studies (16, 17, 18) have confirmed that the efficacy of calcitriol monotherapy in preventing bone loss is inconsistent, with roughly half of the studies showing significant BMD improvements, while the remainder reported neutral outcomes. Although calcitriol was initially assumed to benefit bone through enhanced calcium absorption and PTH suppression, this concept does not apply to PHPT, where calcitriol excess is driven by PTH-dependent 1α-hydroxylase activation rather than compensatory physiology, making its role in PHPT-related osteoporosis more complex and not fully understood. Reid *et al.* (19) demonstrated in a large retrospective cohort (*n* = 611) that osteoporosis in PHPT was predicted mainly by demographic rather than biochemical factors, as age and BMI – but not PTH or calcium – remained significant in multivariate analysis. This supports the view that standard biochemical markers insufficiently capture the skeletal consequences of PHPT. In contrast, our study identifies serum calcitriol as an independent predictor of osteoporosis, suggesting that chronic activation of vitamin D metabolism may better reflect cumulative skeletal exposure to PTH overactivity than single-time-point PTH measurements.

Christensen *et al.* (20) investigated the relationships between vitamin D metabolites, PTH, and BMD in 52 patients with confirmed PHPT. They demonstrated that higher serum calcitriol levels were negatively associated with distal radius BMD, whereas PTH lost significance in multivariate analysis. No correlation between calcitriol and 25(OH)D was also observed. These findings support the concept that calcitriol may serve as a more stable indicator of the chronic skeletal effects of PTH overstimulation than PTH itself and that the conversion to the active metabolite may depend more on renal hydroxylation than on substrate availability. However, in contrast to our cohort – where calcitriol was associated with the overall presence of osteoporosis rather than site-specific bone loss – the Danish study identified this relationship primarily at cortical bone sites, suggesting possible differences in bone compartment vulnerability or disease phenotype between study populations. These findings are consistent with those reported by Moosgaard *et al.* (11) who demonstrated that circulating calcitriol levels in PHPT are largely independent of vitamin D status and PTH concentrations, being primarily determined by renal function and phosphate handling. In line with their results, our study also found no correlation between serum 1,25(OH)_2_D and 25(OH)D, supporting the hypothesis that calcitriol synthesis reflects chronic renal 1α-hydroxylase activity rather than substrate availability. Moreover, in our cohort, calcitriol – but not PTH – remained an independent predictor of osteoporosis, supporting the concept that downstream markers of sustained PTH activity may better capture cumulative skeletal effects than circulating PTH levels alone.

Hypophosphatemia and chronic renal phosphate wasting are well-established contributors to impaired bone mineralization and osteoporosis (21, 22). Reduced phosphate availability limits hydroxyapatite formation, leading to defective mineral deposition and osteoid accumulation (23). In PHPT, excess PTH – often amplified by elevated calcitriol – promotes phosphaturia through downregulation of proximal tubular sodium-phosphate (NaPi-IIa/IIc) co-transporters (24). Consistent with this mechanism, TMP/GFR in our cohort correlated inversely with both PTH and calcitriol. Previous studies have shown that phosphate wasting is common in PHPT and is associated with preferential cortical bone loss, supporting its role in the skeletal phenotype of the disease (25). Our results extend these observations by demonstrating that lower TMP/GFR, as an integrated marker of PTH-mediated phosphate wasting, is independently associated with osteoporosis. Large PHPT cohorts have similarly reported associations between hypophosphatemia, osteoporosis, and more severe disease, while highlighting the limited discriminatory value of serum phosphate alone (26, 27). Taken together, these findings suggest that TMP/GFR may capture aspects of phosphate availability relevant to skeletal health that are not reflected by static serum or urinary measurements. From a clinical perspective, TMP/GFR is not intended as a routine stand-alone test but may be realistically applied as an adjunctive, context-dependent parameter in selected PHPT patients to refine skeletal risk assessment when conventional markers yield inconclusive information.

The relationship between nephrolithiasis and osteoporosis remains uncertain and incompletely understood. A meta-analysis by Lucato *et al.* and a large cumulative analysis by Jia *et al.* demonstrated a higher prevalence of osteoporosis among stone formers in the general population, with effect modification by age and sex (28, 29). More recently, NHANES-based analyses combined with bioinformatic approaches confirmed an independent but sex-dependent association, with stronger and more consistent effects observed in women than in men (30). Taken together, these data indicate that any link between nephrolithiasis and skeletal health in the general population is modest, context-dependent, and strongly influenced by demographic factors rather than reflecting a uniform or causal relationship. In contrast to these population-based observations, all cases of nephrolithiasis in our cohort occurred exclusively in patients with osteoporosis. This observation is consistent with previous studies indicating that, in primary hyperparathyroidism, nephrolithiasis often co-occurs with skeletal involvement and likely reflects shared underlying metabolic disturbances rather than isolated organ-specific pathology (1, 31). In PHPT, PTH excess drives hypercalciuria, accelerated bone turnover, negative phosphate balance, and calcitriol excess, leading to simultaneous skeletal demineralization and renal stone formation. It distinguishes this relationship from that observed in the general population. Furthermore, our cohort was characterized by a marked female predominance, a group in whom both osteoporosis and stone-related skeletal associations are more pronounced. Although age correlated positively with osteoporosis and inversely with renal function in univariate analyses, these associations did not persist in multivariate models, suggesting that biochemical disturbances intrinsic to PHPT may outweigh demographic factors in determining skeletal risk. Nevertheless, these findings should be interpreted with caution and warrant confirmation in larger, multicenter PHPT cohorts.

The Cl/Pi ratio has been proposed as a biochemical marker that reflects the biochemical signature of phosphate excretion and serving as an indirect indicator of altered phosphate homeostasis in PHPT (32). Wang *et al.* demonstrated that Cl/Pi correlates with PTH and calcium and can help discriminate PHPT from controls, especially when combined with alkaline phosphatase (33). In our cohort, Cl/Pi showed a similar biochemical behavior, correlating positively with PTH and calcitriol and strongly inversely with TMP/GFR, indicating that it reflects PTH-driven phosphate wasting. However, unlike TMP/GFR, Cl/Pi was not associated with osteoporosis or nephrolithiasis, indicating that it reflects biochemical disease activity rather than clinical outcomes.

This study has several limitations. First, its cross-sectional design does not allow for causal inference or evaluation of longitudinal changes in bone density or fracture risk. Moreover, the sample size, although comparable to other PHPT cohorts, may limit subgroup analyses and the generalizability of the findings. We also did not assess fibroblast-growth-factor-23 (FGF-23), a key regulator of phosphate metabolism (34), which could provide additional insight into phosphate regulation. Finally, single-time-point biochemical measurements may not fully account for the variability of serum phosphate or urinary indices, underscoring the need for larger prospective studies to validate the predictive role of TMP/GFR and calcitriol. Finally, the inclusion of patients deemed ineligible for surgery may represent a potential selection bias, as this group may differ clinically from patients referred for parathyroidectomy. However, eligibility was assessed according to current international guidelines, reflecting real-world clinical decision-making.

In this study, we identified serum calcitriol and TMP/GFR as independent predictors of osteoporosis in patients with PHPT, reflecting downstream effects of chronic PTH excess rather than mechanisms acting beyond PTH itself. TMP/GFR outperformed serum phosphate as a marker of phosphate availability and may serve as a more informative adjunctive parameter in selected PHPT patients when conventional biochemical markers yield inconclusive information.

## Declaration of interest

The authors declare that there is no conflict of interest that could be perceived as prejudicing the impartiality of the work reported.

## Funding

The authors declare that no funds, grants, or other support were received during the preparation of this manuscript.

## Author contribution statement

SS and MP conceived the study and performed formal analysis and investigation. SS curated the data and wrote the original draft of the manuscript. MP designed the methodology. MP and UA supervised the study and administered the project. UA provided resources. SS, MP, and UA performed validation and visualization and reviewed and edited the manuscript. All authors have read and agreed to the published version of the manuscript.

## Data availability

The data of this study is available on request from the corresponding author.

## Institutional review board statement

The study was approved by the Commission of Bioethics at the Medical University of Warsaw, Poland (AKBE 136/2025; May 12, 2025), and conducted with the written consent of all participants, in accordance with the Declaration of Helsinki.

## Informed consent statement

Due to the retrospective and non-interventional design of the study involving only anonymized medical data, informed consent was not required.

## Use of AI tools

Minor assistance from an AI-based language tool (ChatGPT, GPT-5 model, OpenAI) was used for grammar and style editing only. All scientific content, data analysis, and interpretations were prepared entirely by the authors.
